# Hematologic and lymphatic system toxicities associated with immune checkpoint inhibitors: a real-world study

**DOI:** 10.3389/fphar.2023.1213608

**Published:** 2023-10-31

**Authors:** Na Li, Yong Feng, XiaoLing Chen, Ye Li, Chengmiao Zhang, Yin Yin

**Affiliations:** ^1^ Department of Central Laboratory, Shenyang Tenth People’s Hospital, Shenyang Chest Hospital, Shenyang, China; ^2^ Department of Thoracic Surgery, Shenyang Tenth People’s Hospital, Shenyang Chest Hospital, Shenyang, China; ^3^ Department of Pathology, Shenyang Tenth People’s Hospital, Shenyang Chest Hospital, Shenyang, China

**Keywords:** immune checkpoint inhibitors, hematologic and lymphatic toxicities, FDA adverse event reporting system, programmed cell death protein 1/programmed cell death ligand 1, cytotoxic T-lymphocyte-associated protein, monotherapy, bevacizumab, combination therapy

## Abstract

**Introduction:** Immune checkpoint inhibitors (ICIs) exert antitumor responses in many types of cancer but may also induce serious or fatal toxicities that affect all organ systems, including the hematologic and lymphatic systems. However, the risk of hematologic and lymphatic system toxicities following different ICI treatments remains unknown. This study aimed to describe the hematologic and lymphatic system toxicities associated with different ICI regimens and the impact of combining ICIs with anti-vascular endothelial growth factor drugs using the United States Food and Drug Administration Adverse Event Reporting System pharmacovigilance database.

**Methods:** The reporting odds ratio (ROR) and information component (IC) indices were used to identify disproportionate reporting of ICI-associated hematologic and lymphatic adverse events (AEs).

**Results:** We extracted 10,971 ICI-associated hematologic and lymphatic AEs from 35,417,155 reports. These AEs were more frequently reported in female patients (ROR: 1.04 95% confidence interval [CI]: 1.01–1.07) and younger patients (ROR: 1.05 95% CI: 1.01–1.09). The disseminated intravascular coagulation fatality rate (63.97%) was the highest among the reported preferred terms, despite its low incidence (3.32%). The time to onset of ICI-related hematologic and lymphatic AEs was relatively short, with 77.44% reported within 3 months. Disproportionate analysis showed that most ICIs were associated with significant overreporting of hematologic and lymphatic AEs (IC_025_: 0.34 and ROR_025_: 2.10). Hematologic and lymphatic system AEs were more frequently reported in patients treated with anti-programmed cell death protein 1/programmed cell death ligand 1 monotherapy than in those treated with anti-cytotoxic T-lymphocyte-associated protein 4 monotherapy (ROR: 1.54, 95% CI: 1.38–1.71), with atezolizumab showing the strongest signal (ROR_025_: 4.19, IC_025_: 1.00). In patients receiving combined treatment, ICIs plus bevacizumab exerted a higher disproportion signal than monotherapy (ROR: 161, 95% CI: 1.75–1.88).

**Discussion:** The spectrum of hematologic and lymphatic AEs differed according to the ICI regimen. Early recognition and management of ICI-related hematologic and lymphatic AEs are vital in clinical practice.

## 1 Introduction

Immune checkpoint inhibitors (ICIs) targeting the programmed cell death protein 1/programmed cell death ligand 1 (PD-1/PD-L1) and cytotoxic T-lymphocyte-associated protein 4 (CTLA-4) have revolutionized the treatment landscape for a wide range of cancers, demonstrating significant efficacy and favorable responses and pioneering a new therapeutic paradigm for many types of solid tumors (Ribas and Wolchok, 2018; Tang et al., 2018). Compared with monotherapy, combined ICI blockade can further improve clinical outcomes (Hammers et al., 2017; Hellmann et al., 2019; Wolchok et al., 2022). To date, three main types of ICIs have been approved by the United States Food and Drug Administration (FDA): PD-1 inhibitors such as cemiplimab, nivolumab, and pembrolizumab; PD-L1 inhibitors such as atezolizumab, avelumab, and durvalumab; and CTLA-4 inhibitors such as ipilimumab and tremelimumab (Ribas and Wolchok, 2018).

Although clinically effective, ICIs can be accompanied by severe and sometimes fatal organ system toxicities, including hematologic and lymphatic toxicities (Kennedy and Salama, 2020; Ghanem et al., 2022). Frequently reported ICI-related hematologic and lymphatic system complications include anemia (Chambers et al., 2022), thrombocytopenia (Xie et al., 2021), and pure red cell aplasia (Wright and Brown, 2017; Kroll et al., 2022). ICI-induced hematologic and lymphatic adverse events (AEs) are rare, with an incidence of 3.6% for all grades and 0.7% for grades III–IV (Michot et al., 2019; Petrelli et al., 2018). However, ICIs can lead to serious and life-threatening AEs, which are reported less frequently than common AEs and have not been extensively characterized.

Vascular endothelial growth factor (VEGF)-dependent angiogenesis plays a critical role in tumorigenesis and progression (Goel and Mercurio, 2013; Ferrara and Adamis, 2016). Bevacizumab, a recombinant humanized immunoglobulin (Ig) G1 monoclonal antibody targeting VEGF, was the first angiogenesis inhibitor approved by the United States FDA for treating a wide range of tumors (Viallard and Larrivée, 2017). VEGF can attenuate the antitumor immune response by reprogramming the tumor immune microenvironment; thus, anti-VEGF combined with immunotherapy has a potential synergistic antitumor effect (Bejarano et al., 2021). However, in combination therapy, the clinical benefits and overlapping toxicity of the drugs must be carefully considered. Although rare, bevacizumab can also cause some hematological complications, such as thrombocytopenia. A phase III randomized trial of bevacizumab for glioblastoma showed a higher incidence of thrombocytopenia than the placebo (34.1% vs. 27.3%) (Saran et al., 2016). According to a case report, a 59-year-old male patient with colon adenocarcinoma developed thrombocytopenia after treatment with bevacizumab (Kumar et al., 2012). Owing to the complex biological effects of combined ICI and VEGF inhibitor use, whether this combination enhances or reduces the toxicities of the hematologic and lymphatic systems remains unclear.

Given the increase in the use of ICIs in clinical practice, the potential risk to the hematologic and lymphatic systems should be considered. Herein, we report the results of a systematic analysis using real-world pharmacovigilance data to investigate the association of hematologic and lymphatic system toxicities of different ICI treatment regimens and further consider the effect of bevacizumab to provide evidence for clinical practice.

## 2 Materials and methods

### 2.1 Data sources and study design

This retrospective, observational pharmacovigilance study was conducted using the FDA Adverse Event Reporting System (FAERS) database, which is a collection of reports of AEs that allows for signal detection and quantification of the association between drugs and reporting of AEs (Min et al., 2018). All variables for each record, including age, sex, outcomes, drug name, reporting year, and reporting country, can be extracted from the FAERS database. AEs were coded using preferred terms (PTs) according to the international Medical Dictionary for Regulatory Activities (MedDRA). A specific PT was assigned to high-level terms and system-organ classes. In addition, we removed duplicate records using FDA’s recommended method by choosing the latest FDA_DT if the CASEID was the same and choosing the higher PRIMARYID if the CASEID and FDA_DT were the same. In this analysis, the coverage period was from 1 January 2014 to 31 December 2022. The studied drugs included anti-PD-1 (nivolumab, cemiplimab, and pembrolizumab), anti-PD-L1 (atezolizumab, avelumab, and durvalumab), anti-CTLA-4 (ipilimumab and tremelimumab), and anti-VEGF antibodies. As FAERS does not use a uniform coding system for medications, both generic and brand-name drugs were used to identify study drug-associated records. The details of the drug names are listed in Supplementary Table S1. This study included both monotherapy and combination therapy. Toxicity was attributed to monotherapy if one drug was reported as the “primary suspect” and to combination therapy if one drug was reported as the “primary suspect” and other drugs were reported as “secondary suspects.” This study included all blood and lymphatic system disorders (MedDRA code: 10005329) according to MedDRA version 25.0.

### 2.2 Statistical analysis

Descriptive analysis was performed to summarize clinical features. Disproportionality analysis was used to evaluate specific AEs associated with a given drug (Ali et al., 2021). Reporting odds ratios (RORs) and information components (ICs) were used as indicators of disproportionality (Ang et al., 2016; Gatti et al., 2021). A significant signal was defined if the lower limit of the 95% confidence interval (CI) of ROR (ROR_025_) was >1 in at least three cases or the lower limit of the 95% CI of the IC (IC_025_) was >0. The equations for the two algorithms are provided in Supplementary Table S2. In our analysis, ICIs were compared with all other drugs in the full database. We did not consider ICIs in combination with chemotherapy owing to the limitations of chemotherapy drug screening. We performed disproportionality analyses for different subgroups, including sex, age, and different therapies (ROR only). Data were analyzed using SAS version 9.4 (SAS Institute Inc., Cary, NC, United States) and Microsoft Office Excel version 2023 (Microsoft Corp., Redmond, WA, United States).

## 3 Results

### 3.1 Identifying hematologic and lymphatic AEs from FAERS

**FIGURE 1:**
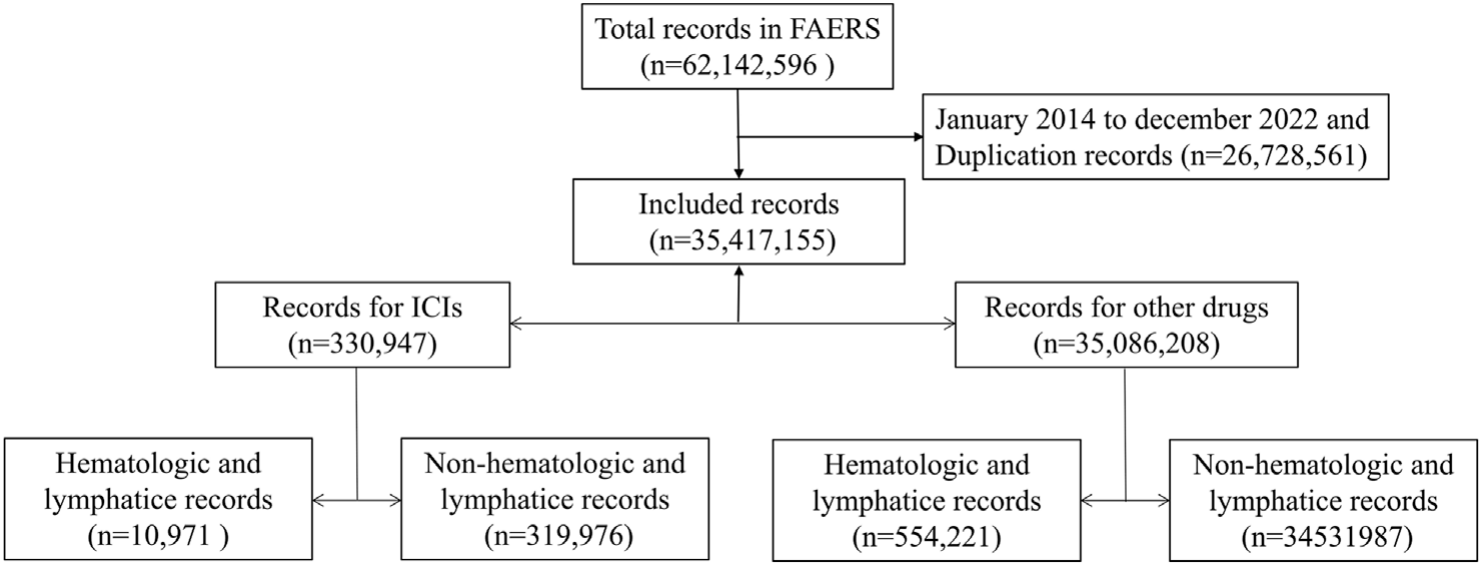
Screening process for adverse event records in the FAERS database.

To date, 62,142,596 records have been deposited in the FAERS database. After excluding duplicate records, 35,417,155 records were selected from 1 January 2014 to 31 December 2022, of which 330,947 were associated with ICI-related AEs. Subsequently, 10,971 records were screened for hematologic and lymphatic AEs associated with ICIs. In addition, 554,221 records on hematologic and lymphatic AEs associated with other drugs were included in the analytic dataset (Figure 1).

### 3.2 Descriptive analysis from FAERS

The clinical characteristics of patients with ICI-induced hematologic and lymphatic AEs are listed in Table 1. We found that 83.38% of the cases were reported in 2018–2022, reflecting a substantial increase in the use of ICIs in recent years. Most reports were from Japan (3,045, 27.75%), the United States (2,267, 20.66%), and France (1,143, 10.42%). There were more reports on men (55.37%) than on women (37.00%) and on patients aged ≥65 years (44.11%) than on those aged <65 years (36.25%).

**TABLE 1:** Clinical characteristics of patients with ICI- and other drug-induced hematological and lymphatic system toxicity.

Characteristics	Hematological and lymphatic system AEs with ICIs (*n* = 10971)	Hematological and lymphatic system AEs with other drugs (*n* = 554221)
Sex		
Female	4059 (37.00%)	262811 (47.42%)
Male	6075 (55.37%)	217088 (39.17%)
Missing	837 (7.63%)	74321 (13.41%)
Age, years		
<65	3977 (36.25%)	240865 (43.46%)
≥65	4839 (44.11%)	164826 (29.74%)
Missing	2155 (19.64%)	148529 (26.80%)
Reporting year		
2014	110 (1.00%)	19398 (3.50%)
2015	299 (2.73%)	23832 (4.30%)
2016	507 (4.62%)	43783 (7.90%)
2017	907 (8.27%)	36024 (6.50%)
2018	1242 (11.32%)	55422 (10.00%)
2019	1583 (14.43%)	65952 (11.90%)
2020	1740 (15.86)	92001 (16.60%)
2021	2094 (19.09%)	104194 (18.80%)
2022	2489 (22.69%)	113615 (20.50%)
Reporting country		
United states	2267 (20.66%)	212322 (38.31%)
Japan	3045 (27.75%)	42120 (7.60%)
France	1143 (10.42%)	46665 (8.42%)
Germany	769 (7.01%)	30371 (5.48%)
China	638 (5.82%)	19397 (3.50%)
Italy	476 (4.34%)	23831 (4.30%)
Others	2017 (18.38%)	135728 (24.49%)
Missing	616 (5.61%)	101533 (18.32%)
Outcome		
Death	2180 (19.87%)	49935 (9.01%)
Hospitalization	4519 (41.19%)	189710 (34.23%)
Other serious events	3643 (33.20%)	271900 (49.06%)
Life threatening	203 (1.85%)	33807 (6.10%)
Disability	160 (1.46%)	6816 (1.23%)
Required	5 (0.05%)	1109 (0.20%)
Missing	189 (1.72%)	943 (0.17%)

ICIs, immune checkpoint inhibitors.

Data are presented as *n* (%).

**FIGURE 2:**
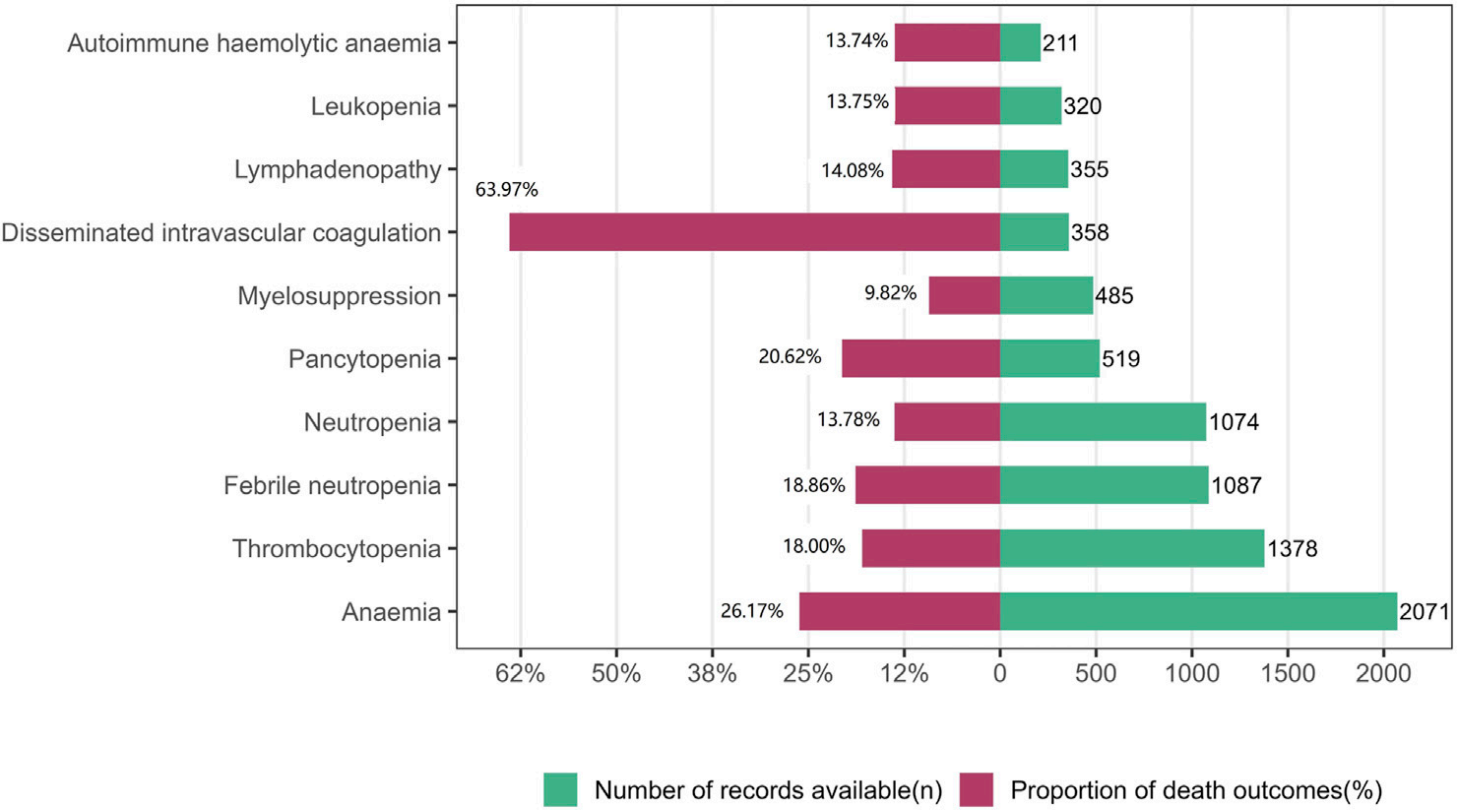
Records and proportions of deaths in class-specific hematological and lymphatic system adverse events.

In the analysis of deaths due to the 10 most frequently reported PTs in class-specific hematologic and lymphatic AEs, 2,180 (19.87%) were associated with ICIs (Table 1). Further analyses revealed that the severity of these events varied. In general, anemia, thrombocytopenia, febrile neutropenia, neutropenia, pancytopenia, myelosuppression, disseminated intravascular coagulation, lymphadenopathy, leukopenia, and autoimmune hemolytic anemia were the 10 most frequently reported PTs in class-specific hematologic and lymphatic systems. Although the incidence of disseminated intravascular coagulation (358/10,791%, 3.32%) was low, its case fatality rate (229/358%, 63.97%) was the highest among the 10 most frequently reported PTs. Anemia had the highest incidence (2,071/10,791%, 19.19%) and the second highest case fatality rate (542/2,071%, 26.17%; Figure 2).

### 3.3 Time to onset (TTO)

**FIGURE 3:**
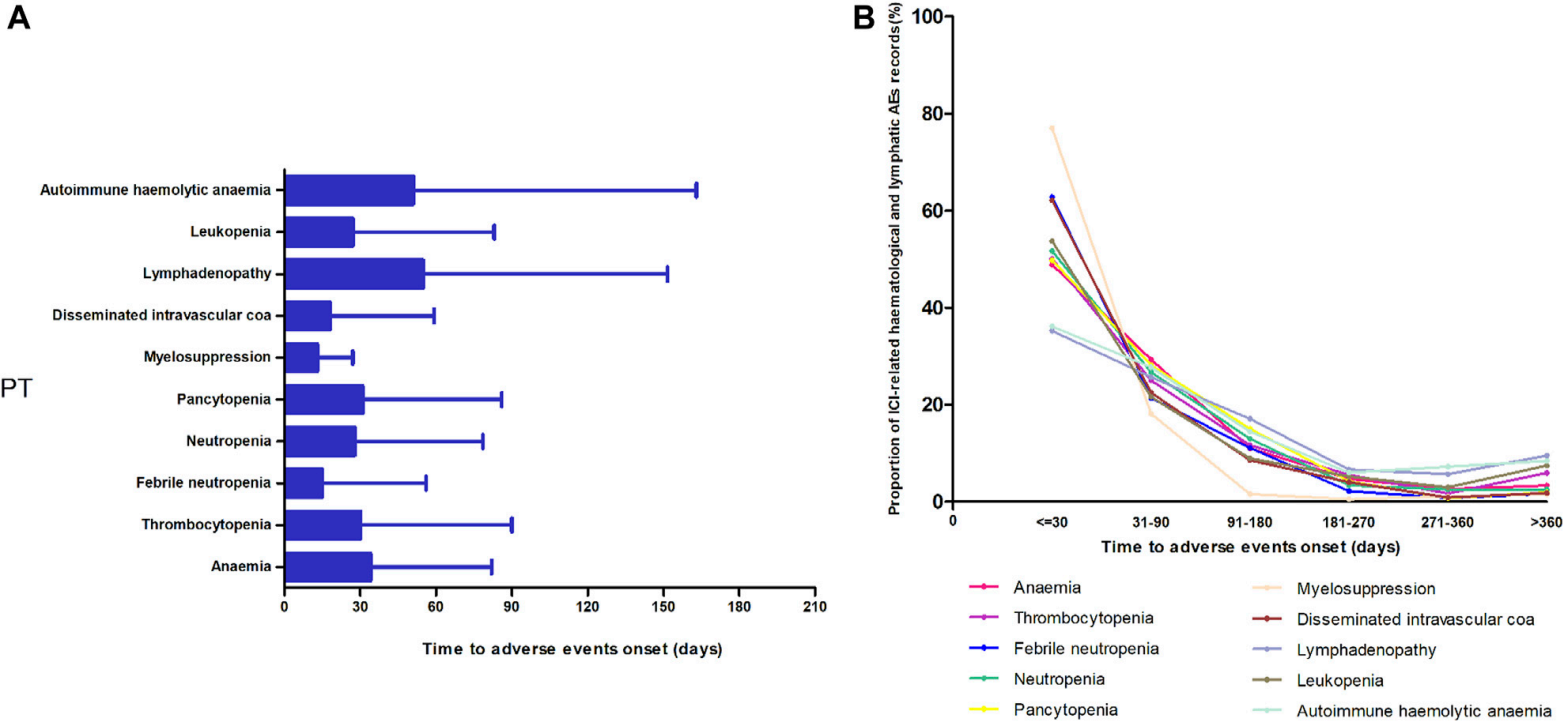
Time of onset of the top 10 most frequently reported preferred terms in the hematological and lymphatic systems. **(A)** Time of onset and **(B)** the cumulative proportion at different periods.

Figure 3 shows the TTO of the 10 most frequently reported PTs in the ICI-related hematologic and lymphatic systems. After excluding records with no event times, 5,417 records were included. A higher cumulative proportion of ICI-related hematologic and lymphatic AE records occurred 1 month after administration (51.36%, 2,782/5,417) than at any other time point. Of the ICI-related hematologic and lymphatic AEs, 77.44% (4,195/5,417) occurred within 3 months. Overall, the data for myelosuppression showed a relatively short median onset time (13 days), whereas those for lymphadenopathy and autohemolytic anemia had relatively long median onset times of 55 and 51 days, respectively (Figure 3).

### 3.4 Disproportionality analysis

The signal values and associations between hematologic and lymphatic AEs and different ICI regimens are summarized in Table 2. In general, higher reporting frequencies of hematologic and lymphatic AEs were observed for most ICI regimens. The ROR_025_ was 2.10 and IC_025_ was 0.35 for ICIs, compared to the whole database. AEs were reported more frequently in female patients than in male patients (ROR: 1.05, 95% CI: 1.01–1.08, Supplementary Table S3) and in patients aged <65 years than in those aged ≥65 years (ROR: 1.05, 95% CI: 1.01–1.09, Supplementary Table S3). Regarding monotherapy, most hematologic and lymphatic AEs were reported for anti-PD-1 agents (*N* = 6407, 58.40%), whereas anti-PD-L1 drugs contributed to a lower proportion of AEs (*N* = 1975, 18.00%) but had stronger signal values (ROR_025_: 7.68, IC_025_: 2.84), especially atezolizumab, which had the strongest signal values (ROR_025_: 4.19, IC_025_: 1.00) among the monotherapies reported.

**TABLE 2:** Signal value of hematological and lymphatic system AEs associated with different immunotherapy regimens.

Strategy	a (N)	b	c	d	ROR	ROR_025_	ROR_975_	IC	IC_025_	IC_975_
**Total**	10971	319976	554221	34531987	2.14	**2.10**	2.18	1.05	**0.35**	1.75
ICIs										
Nivolumab	3343	108806	561849	34743157	1.90	**1.84**	1.97	0.9	**0.05**	1.75
Pembrolizumab	2961	87965	562231	34763998	2.08	**2.00**	2.16	1.03	**0.16**	1.90
Cemiplimab	103	3024	565089	34848939	2.10	**1.73**	2.56	1.04	−0.48	2.56
Atezolizumab	1379	20329	563813	34831634	4.19	**3.97**	4.43	1.99	**1.00**	2.98
Durvalumab	508	11878	564684	34840085	2.64	**2.41**	2.88	1.36	**0.19**	2.53
Avelumab	88	3109	565104	34848854	1.75	**1.42**	2.16	0.78	−0.78	2.34
Ipilimumab	372	16050	564820	34835913	1.43	**1.29**	1.58	0.50	−0.73	1.73
Polytherapy1	1476	54518	563716	34797445	1.67	**1.59**	1.769	0.72	−0.25	1.64
Polytherapy2	36	1430	565156	34850533	1.55	**1.12**	2.16	0.61	−1.20	2.42
Polytherapy3	73	1206	565119	34850757	3.73	**2.95**	4.73	1.81	**0.20**	3.41
ICIs + bevacizumab	753	12615	564439	34839348	3.68	**3.42**	3.96	1.81	**0.72**	2.90
Anti-PD-1/PD-L1 vs. anti-CTLA-4	8382	235111	372	16050	1.54	**1.38**	1.71			
Polytherapy vs. monotherapy	1559	57489	8754	251161	0.77	0.74	0.82			
ICIs + bevacizumab vs. ICIs	753	12615	10971	319976	1.74	**1.61**	1.88			

Note: Bold text denotes significant signals.

ICIs, immune checkpoint inhibitors; Polytherapy1, nivolumab + ipilimumab; Polytherapy2, nivolumab + pembrolimab + ipilimumab; Polytherapy3, durvalumab + tremelimumab; CI, confidence interval; ROR, reporting odds ratio; ROR_025_, lower limit of the 95% two-sided CI, of the ROR; ROR_075_, upper limit of the 95% two-sided CI, of the ROR. a(N), the number of reports containing both ICIs, and hematological and lymphatic system AEs, in one subgroup; b, the number of reports containing both ICIs, and all other adverse events (except hematological and lymphatic system AEs) in one subgroup; c, the number of reports containing both ICIs, and hematological and lymphatic system AEs, in another subgroup; and d, the number of reports containing both ICIs, and all other adverse events in another subgroup.

Hematologic and lymphatic toxicities were more frequently reported in patients treated with anti-PD-1/PD-L1 than in those treated with anti-CTLA-4 (ROR_025_: 1.38). Among the combination therapies, nivolumab plus ipilimumab was the most common (*N* = 1476, 13.45%), but only its ROR was significant (ROR_025_: 1.59, IC_025_: −0.25). Durvalumab plus tremelimumab showed a stronger signal (ROR_025_: 2.95, IC_025_: 0.20) than the above group, despite contributing a very small proportion of reported AEs (*N* = 73, 0.06%). Furthermore, ICIs plus anti-VEGF therapy showed the highest signal value among the combined therapies (ROR_025_: 3.42, IC_025_: 0.72). Disproportionate reporting was also found in combination therapy compared with monotherapy: hematologic and lymphatic AEs were more frequently reported in patients treated with ICI plus anti-VEGF combination therapy than in those treated with ICI monotherapies (ROR: 1.74, 95% CI: 1.61–1.88).

### 3.5 Spectrum of hematologic and lymphatic AEs in different ICI regimens

**FIGURE 4:**
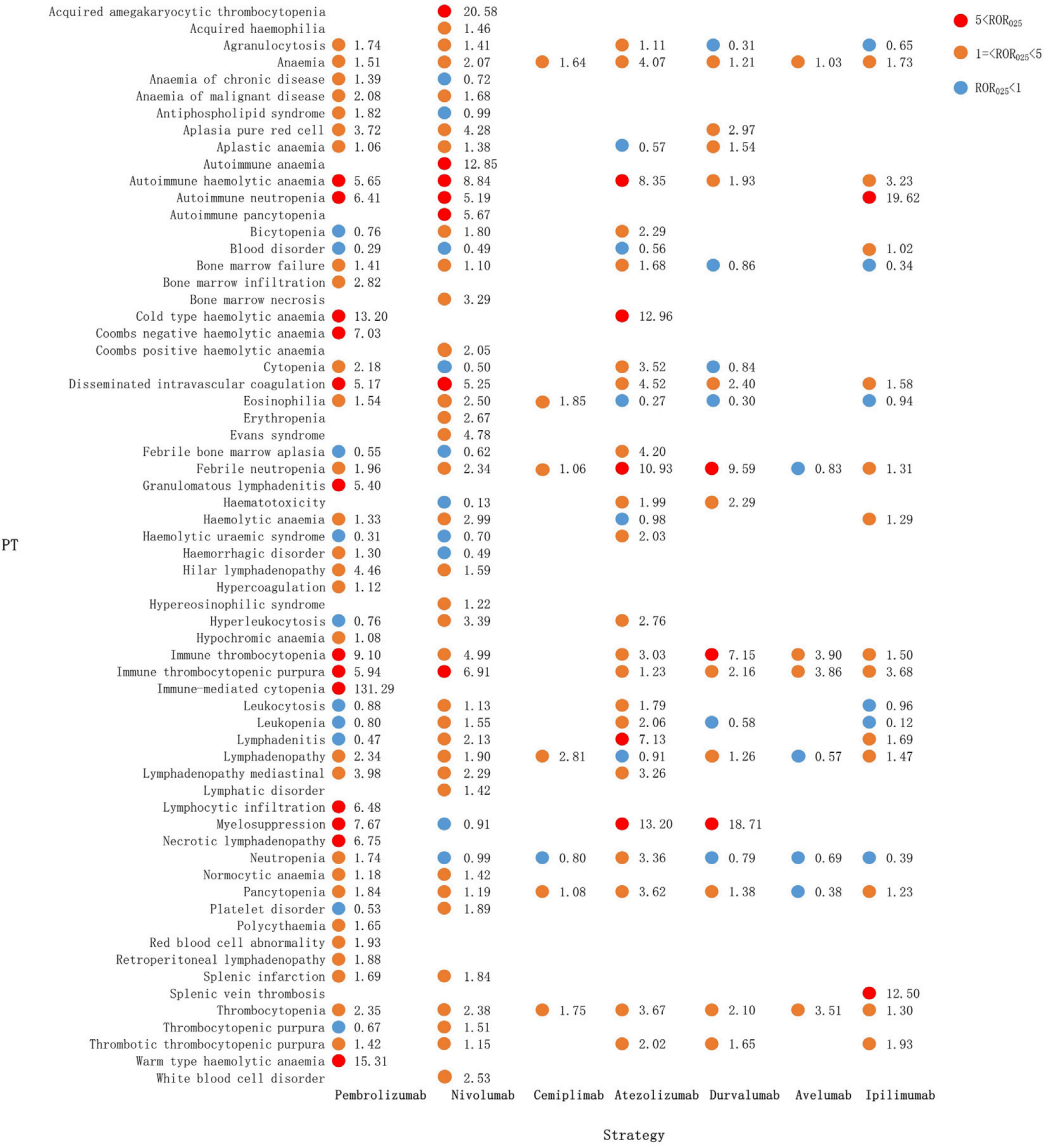
Hematological and lymphatic system toxicities for different ICI monotherapy strategies. PT, preferred term; IC, information component; IC_025_, lower limit of the 95% confidence interval of IC; IC_025_, greater than 0 was deemed a signal; ICI, immune checkpoint inhibitor.

Figure 4 shows the hematologic and lymphatic system toxicity profiles of the different ICI monotherapy regimens. A total of 59 class-specific signals were significant in the anti-PD-1/PD-L1 classes compared with 15 signals in the anti-CTLA-4 class. Among the PD-1 inhibitors, pembrolizumab showed the broadest spectrum of hematologic and lymphatic AEs, with 41 PTs detected as signals, ranging from aplastic anemia (ROR_025_: 1.06) to immune-mediated cytopenia (ROR_025_: 131.29). There were 40 PTs significantly associated with nivolumab treatment, ranging from bone marrow failure (ROR_025_: 1.10) to acquired amegakaryocytic thrombocytopenia (ROR_025_: 20.58). There were six PTs significantly associated with cemiplimab treatment, ranging from febrile neutropenia (ROR_025_: 1.06) to lymphadenopathy (ROR_025_: 2.81). These six PTs overlapped with those of pembrolizumab and nivolumab therapy and included lymphadenopathy, eosinophilia, thrombocytopenia, anemia, pancytopenia, and febrile neutropenia.

The hematologic and lymphatic system spectra of anti-PD-L1 drugs varied substantially, with 24 PTs significantly associated with the atezolizumab treatment, ranging from agranulocytosis (ROR_025_: 1.11) to myelosuppression (ROR_025_: 13.20). There were 14 PTs associated with durvalumab, ranging from anemia (ROR025: 1.21) to myelosuppression (ROR025: 18.71). The following PTs were uniquely associated with atezolizumab: pure red cell aplasia, aplastic anemia, and lymphadenopathy. For the anti-PD-L1 group, myelosuppression was the most significant signal associated with atezolizumab (ROR_025_: 13.20) and durvalumab (ROR_025_: 18.71), followed by febrile neutropenia (ROR_025_: 10.93 and 9.59, respectively). The four PT signals detected by avelumab all overlapped with atezolizumab and durvalumab. Anti-CTLA-4 treatment had 15 PTs significantly associated with ipilimumab, with 13 PTs overlapping with anti-PD-1 and 12 PTs with anti-PD-L1 (Figure 4).

**FIGURE 5:**
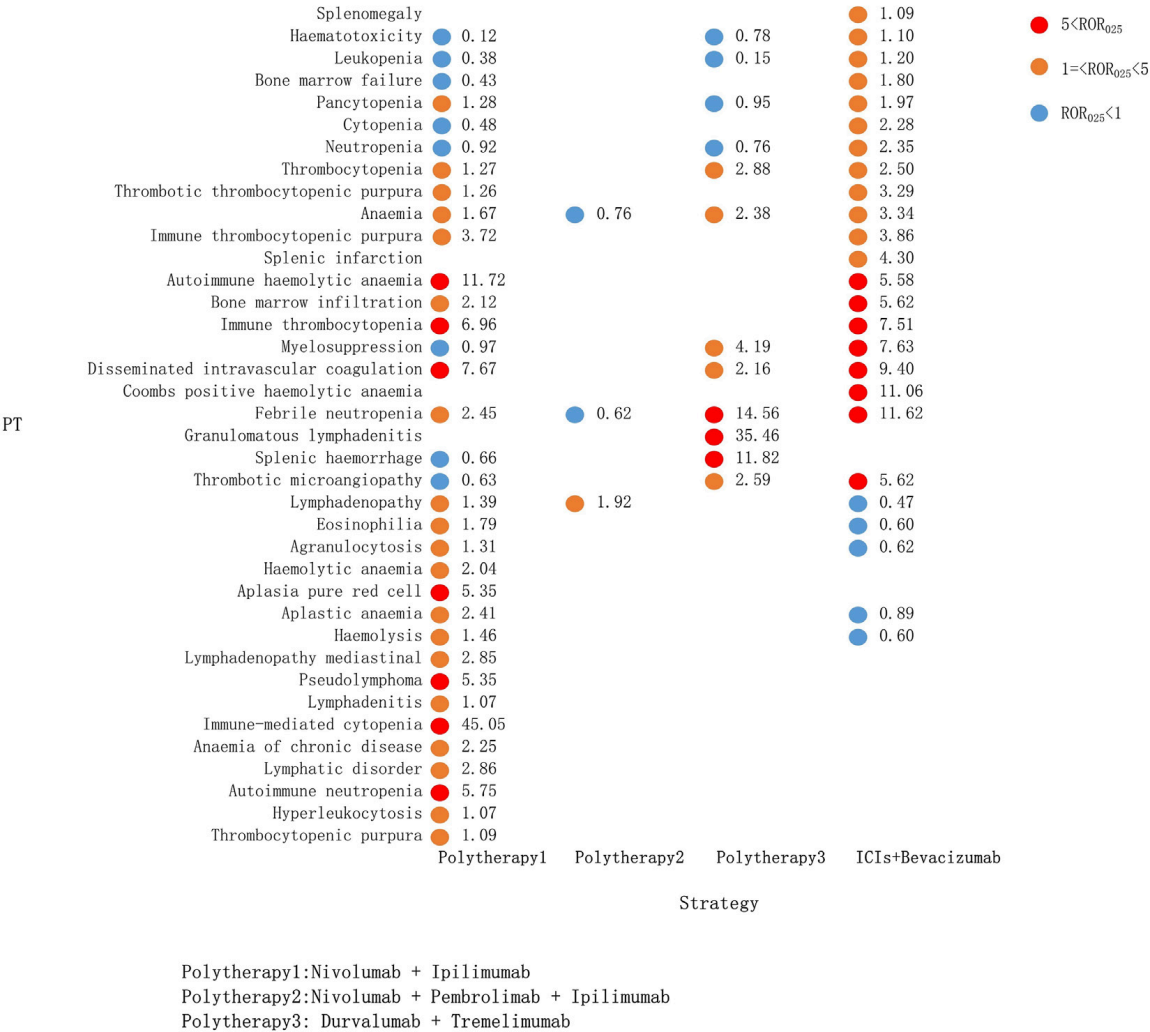
Hematological and lymphatic system toxicities for different ICI combination therapy strategies. PT, preferred term; IC, information component; IC_025_, the lower end of the 95% confidence interval of IC; IC_025_, greater than 0 was deemed a signal; ICI, immune checkpoint inhibitor.

Compared with the immune-monotherapy group, the double-ICI blockade group had relatively few PTs: 33 class-specific signals were detected, of which 4 were newly generated, namely, hemolysis, pseudolymphoma, thrombotic microangiopathy, and splenic hemorrhage. Notably, splenic hemorrhage had a relatively high signal for the durvalumab plus tremelimumab treatment (ROR_025_: 11.82). Nivolumab plus ipilimumab, the most common tumor treatment, showed the broadest spectrum of hematologic and lymphatic system diseases, ranging from lymphadenitis (ROR_025_: 1.07) to immune-mediated cytopenia (ROR_025_: 45.05). As mentioned above, immune-mediated cytopenia was also the strongest signal in pembrolizumab monotherapy. In ICI plus bevacizumab treatment, 19 PT signals were detected, 5 of which were not present in the other combination regimens (Figure 5).

**FIGURE 6:**
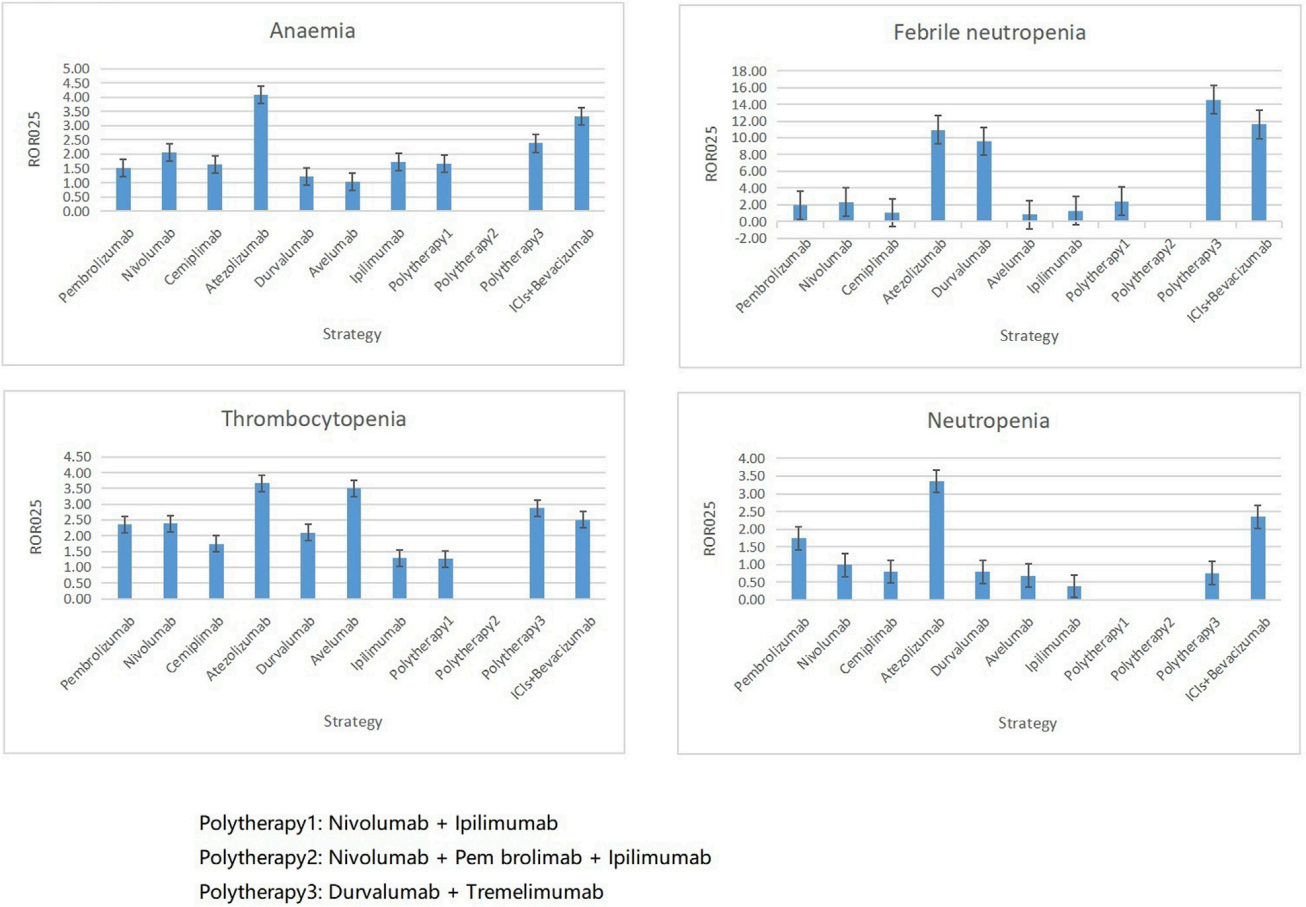
Associations between four top ranked PTs and different ICI strategies. PT, preferred term; IC, information component; ROR_025_, greater than 0 was deemed a signal; ICI, immune checkpoint inhibitor.

Overall, anemia (*n* = 207, 19.19%), thrombocytopenia (*n* = 1378, 12.77%), febrile neutropenia (*n* = 1078, 10.07%), and neutropenia (*n* = 1074, 9.95%) were the four most common hematologic and lymphatic system complications in patients who received ICIs. However, their correlation with different ICI therapies varied. Anemia and thrombocytopenia appeared to be associated with the most regimens except polytherapy2 (Figure 6). Febrile neutropenia was strongly associated with atezolizumab plus tremelimumab and ICI plus bevacizumab combination regimens but showed no correlation with avelumab. Similarly, neutropenia was associated with pembrolizumab, atezolizumab, and ICI plus bevacizumab combination regimens only.

## 4 Discussion

ICIs have remarkable clinical benefits against multiple tumor types. Although complications are rare, ICIs can induce various hematologic and lymphatic complications (Delanoy et al., 2019). However, the risk of experiencing hematologic and lymphatic AEs following ICI use has not been clearly quantified. To the best of our knowledge, this is the largest and most comprehensive pharmacovigilance study of ICI-induced hematologic and lymphatic system toxicities to date. In this study, several key findings were noted, and the combination of ICIs with anti-VEGF therapy was considered.

The reporting frequency of ICI-related hematologic and lymphatic AEs was higher in female patients than in male patients. This result is consistent with that of a previous study by Ye et al. (2020). We attribute this to the fact that women tend to have stronger triggered and sustained immune responses against infections and have an increased propensity to develop autoimmune diseases compared to men (Grassadonia et al., 2018). Moreover, in the general population, there are differences in physiological factors, hormone levels, and hemoglobin levels between men and women, and women may be more susceptible to hematologic disorders (Rushton and Barth, 2010). However, the precise factors responsible for sex-related differences remain unclear and require further verification.

The reporting frequency of ICI-related hematologic and lymphatic AEs was also higher in younger patients than in older patients. A correlation between immune-related AEs (irAEs) and age has been hypothesized; however, different studies have yielded conflicting evidence. Conversely, immune senescence increases the risk of serious irAEs in older patients with cancer through an inflammatory process. In 2018, 23,586 FDA safety reports for ICI drugs were analyzed, and data were grouped according to age (<65, 67–75, and >75 years old). In patients with cancer, the incidence of irAEs was higher in those aged ≥65 years than in those aged <65 years for all single agents except atezolizumab (Elias et al., 2018). Another study reported that lower immunity in older patients may result in a lower effect of ICIs and may reduce the occurrence of immune-related AEs (Marur et al., 2018). Therefore, age should be considered in future studies, especially in studies of AEs related to the hematologic system.

The reported case fatality rate due to ICI-related hematologic and lymphatic AEs was higher owing to other drug-induced hematologic and lymphatic AEs, indicating that ICI-related hematologic and lymphatic AEs substantially affect patient mortality. Further analysis showed that the incidence of disseminated intravascular coagulation (DIC) was low, but the case fatality rate was high. DIC is a clinicopathological syndrome and is the most common pathway for the development of many disorders that cause a coagulation dysfunction. Multi-organ dysfunction syndrome is the leading cause of death in patients with DIC, and the death rate due to DIC is 31%–80% (Levi and Sivapalaratnam, 2018). Several cases of DIC associated with ICI therapy have been reported (Alberti et al., 2020; Maiorano et al., 2022). Although rare, in view of the high case fatality rate, it is important to pay close attention to the signs and symptoms of DIC during ICI therapy. In addition, unexpectedly, anemia had the second highest fatality rate. In fact, the high fatality of anemia is associated with the fatal events that it causes. For example, it can lead to anemic heart disease (Goel et al., 2021), heart failure (Chopra and Anker, 2020; Loncar et al., 2021) and acute kidney failure (Locatelli et al., 2021). Furthermore, anemia exacerbates tumor hypoxia, which not only produces proteomic alterations that affect tumor dissemination and lead to malignant progression but also affects the efficacy of various antitumor therapies (Gilreath et al., 2014). Studies have shown that tumor-associated anemia increases the overall risk of death in cancer patients by 65%. Therefore, although anemia is a common complication, clinicians should not ignore it in their practice.

In the TTO analysis, the median TTO of ICI-related hematologic and lymphatic AEs was 28 days, and 77.44% of the events occurred within 3 months. Patients with myelosuppression had the shortest median TTO, and those with lymphadenopathy had the longest. Myelosuppression is the most common side effect of traditional chemotherapy drugs and can also be caused by newer antitumor drugs, such as targeted and immune drugs. Furthermore, more than 80% of chemotherapeutic drugs can lead to myelosuppression, which is mainly caused by central granulocytopenia and thrombocytopenia (Barreto et al., 2014; Fan et al., 2017; Weycker et al., 2019). The incidence of myelosuppression caused by targeted therapy and immunotherapy is significantly lower than that caused by chemotherapy. In addition, there are differences in the mechanisms of action. The onset of myelosuppression due to chemotherapeutic agents usually begins 5–7 days after the end of chemotherapy, peaks at 11–12 days, and then decreases (Wu et al., 2010). This is approximately the same as the time in our study using immunosuppressive agents to cause myelosuppression, suggesting that blood cell levels should be monitored timely, regardless of the treatment regimen used.

Lymphadenopathy is a common disease that can occur at any age and can be benign or malignant. Most cases of superficial lymph node enlargement are caused by non-specific acute/chronic inflammation, reactive hyperplasia, and specific infections. In general, tumors cause only a minority of lymphadenopathies (Maini and Nagalli, 2023). In cancer patients, lymph node enlargement may indicate the presence of local metastasis or disease progression. Meanwhile, pseudoprogression with ICIs may also show lymph node enlargement, which is mainly due to the activation of lymphocytes by ICIs, which causes a large number of lymphocytes to gather in the lymph node area to fight against tumor cells (Borcoman et al., 2019; Guan et al., 2022). Therefore, it is particularly important to clarify the nature of lymph node enlargement in oncological treatment, which is also the key for further selection of treatment options.

Our study assessed and compared the signal intensities of hematologic and lymphatic AEs associated with different ICI regimens. First, we compared ICI regimens with high-frequency AEs reported from the whole database. ICI treatment strategies are associated with a high incidence of toxicity in multiple organ systems, which are not limited to hematologic and lymphatic systems but also include the endocrine (Zhai et al., 2019), respiratory (Cui et al., 2022), hepatic (Remash et al., 2021), and renal systems (Hu et al., 2021). In our study, hematologic and lymphatic AEs were reported more frequently in patients treated with anti-PD-1/PD-L1 monotherapy than in those treated with anti-CTLA-4 monotherapy, with atezolizumab showing the strongest risk signal. Notably, similar trends have been observed in neurological (Haugh et al., 2020) and renal AEs (Hu et al., 2021). A previous study (Michot et al., 2019) reviewed hematologic immune-related AEs with ICIs and reported that the frequency of hematologic AEs of all grades was higher with PD-1 (4.1%) and PD-L1 (4.7%) than with CTLA-4 (0.5%), consistent with our results. However, the precise mechanisms underlying these differences remain unclear and require further investigation.

Our study also provides information on the spectrum of hematologic and lymphatic AEs induced by different ICI regimens and found that the spectra differed according to the treatment regimen. Immune-mediated cytopenia showed the strongest disproportionate signal with pembrolizumab. In 2018, four cases of cytopenia following treatment with ICIs were reported in Texas (Sun et al., 2018). All four cases responded to conventional steroid therapy. Lymphadenopathy, eosinophilia, thrombocytopenia, anemia, pancytopenia, and febrile neutropenia were common to all three PD-1 drugs. Moreover, febrile neutropenia has been linked with pembrolizumab (Tozuka et al., 2018) and nivolumab (Ramchandren et al., 2019) immunotherapy; however, none of the disproportionality signals were statistically significant. In addition, we were unable to find any previous report of an association between cemiplimab and febrile neutropenia. Anemia is a common AE. A systematic review of AEs associated with PD-1 and PD-L1 inhibitor therapy in clinical trials showed that the incidence of anemia as a grade 3 or higher AE was 0.78% (Wang et al., 2019). Myelosuppression showed the most significant disproportionality in PD-L1 monotherapy; however, we were unable to find any published clinical case reports. In the analysis of ICI combination therapy, we identified four signals that have not been reported in the literature previously: hemolysis, pseudolymphoma, thrombotic microangiopathy, and splenic hemorrhage. These findings highlight the importance of signal detection in FAERS.

Studies have shown that VEGF may reprogramme the tumour immune microenvironment through multiple mechanisms. The combination of bevacizumab therapy with ICI therapy has good antitumor effects, especially in non-small-cell lung (Socinski et al., 2021), hepatocellular (Finn et al., 2020), and colorectal (Mettu et al., 2022) cancers. Our study showed that ICIs plus bevacizumab had the highest signal of disproportionality with respect to hematologic and lymphological AEs and was reported more frequently than for ICIs alone. Bai et al. (2021) found that PD-L1 checkpoint inhibitors combination bevacizumab therapy reduced the risk of pneumonia, respiratory failure and disease progression, while increasing the risk of fever, peripheral neuropathy, nephritis and bone marrow failure. The current data are limited to small prospective studies, and a real-world study with a large sample size is still lacking, especially studies of hematologic complications. The most recent pharmacovigilance analysis of ICIs in combination with bevacizumab showed that bevacizumab was an independent risk factor for interstitial lung disease, hypertension, and gastrointestinal bleeding (Gu et al., 2023), but there was no analysis of hematologic AEs. Thus, our results provide novel evidence for informing clinical practice.

This study had some limitations. First, FAERS is a spontaneous reporting system with multiple sources of data, thus suffering from inconsistent formats, duplication, and missing data. Second, the baseline data in the FAERS database are incomplete. Lastly, we did not consider combination chemotherapy regimens in this study, which may have introduced bias into the results. Nevertheless, our study is a systematic and an in-depth quantification of the potential risks to the hematologic and lymphatic systems for both all ICIs and their specific categories, in combination with bevacizumab. These results could provide valuable evidence for further research and clinical practice.

Overall, hematologic and lymphatic system toxicities were more frequently reported in ICI regimens than in other drug regimens, especially among patients treated with anti-PD-1/anti-PD-L1 agents. Compared with ICI monotherapy, ICI plus bevacizumab was associated with a higher incidence of hematologic and lymphatic AEs. Treatment with different ICI immunotherapies may result in unique and distinct profiles of hematologic and lymphatic AEs, depending on the agents used. Therefore, early recognition and management of ICI-related hematologic and lymphatic AEs are vital in clinical practice.

## Data Availability

The original contributions presented in the study are included in the article/Supplementary Material, further inquiries can be directed to the corresponding author.
