# Supplementary material for: Hematologic and lymphatic system toxicities associated with immune checkpoint inhibitors: a real-world study
Source: Front Pharmacol. 2023 Oct 31;14:1213608. doi: 10.3389/fphar.2023.1213608 (PMC10644043; doi:10.3389/fphar.2023.1213608)
Supplement: Supplementary file 1 [file DataSheet1.docx]

Supplementary Material

Hematologic and Lymphatic System Toxicities Associated with Immune Checkpoint Inhibitors: A Real-World Study

Na Li^*^, Yong Feng, XiaoLing Chen, Ye Li, Chengmiao Zhang, Yin Yin

*** Correspondence:** Na Li: 171772958@qq.com

# Supplementary Tables

**Table 1** Generic and brand names of study drugs used in our study

| **Generic names** | **Brand names** | **Approval year** | **Target** |
| --- | --- | --- | --- |
| Nivolumab | Opdivo, BMS-986298, Opdyta, BMS-936558 | 2014 | PD-1 |
| Pembrolizumab | Keytruda, MK-3475 | 2014 | PD-1 |
| Cemiplimab | Libtayo, SAR439684, REGN2810 | 2018 | PD-1 |
| Atezolizumab | Tecentriq, MPDL3280A | 2016 | PD-L1 |
| Avelumab | Bavencio, MSB0010718C | 2017 | PD-L1 |
| Durvalumab | Imfinzi, MEDI4736 | 2017 | PD-L1 |
| Ipilimumab | Yervoy, BMS-734016 | 2011 | CTLA-4 |
| Tremelimumab | Ticilmumab | - | CTLA-4 |
| Bevacizumab | Avastin |  |  |

PD-1, Programmed Death-1; PD-L1, Programmed Death-L1

**Table 2** Summary of major algorithms used for signal detection

| **Algorithms** | **Equation** | **Criteria** | **Code** |
| --- | --- | --- | --- |
| ROR | ROR=(a/b)/(c/d) | ROR_025_>1,N≥3 | ROR=(a/b)/(c/d) |
|  | 95%CI=eln(ROR)±1.96(1/a+1/b+1/c+1/d)^0.5 |  | ROR_025_ = np.exp(np.log(add['ROR']) - 1.96 * (1. / a + 1. / b + 1. / c + 1. / d) ** 0.5) |
|  |  |  | ROR_975_ = np.exp(np.log(add['ROR']) + 1.96 * (1 / a + 1 / b + 1 / c + 1 / d) ** 0.5) |
| IC | IC=log2((Nobs+0.5)/(Nexp +0.5)) | IC025>0 | IC=log2((Nobs+0.5)/(Nexp +0.5)) |
|  | Nobs=a |  | IC_025_ = IC-3.3*(Nobs +0.5)-1/2-2* (Nobs +0.5)-3/2 |
|  | Nexp= ((a +b)*(a + c))/(a + b + c + d) |  | IC_975_ = IC-3.3*(Nobs +0.5)-1/2+2* (Nobs +0.5)-3/2 |

CI, confidence interval; IC, information component; ROR, reporting odds ratio

**Table 3** Signals of ICI-associated hematological and lymphatic system AEs in the different subgroups of genders and ages

| **Groups**  **(ICIs vs. all other drugs)** | **a (N)** | **b** | **c** | **d** | **ROR**  **(ROR_025_-ROR_975_)** | **IC**  **(IC_025_-IC_975_)** |
| --- | --- | --- | --- | --- | --- | --- |
| Sex |  |  |  |  |  |  |
| Male | 6075 | 179856 | 559117 | 34672107 | 2.19 (2.12–2.26) | 1.99 (1.15–2.83) |
| Female | 4059 | 114970 | 561133 | 34736993 | 2.09 (2.04–2.15) | 1.03 (0.26–1.80) |
| Female vs Male | 4059 | 114970 | 6075 | 179856 | 1.04 (1.01–1.07) |  |
| Age |  |  |  |  |  |  |
| <65 | 3979 | 102821 | 561213 | 34749142 | 2.40 (2.32–2.47) | 1.22 (0.39–2.05) |
| ≥65 | 4838 | 131076 | 560354 | 34720887 | 2.29 (2.22–2.35) | 1.16 (0.35–1.96) |
| <65 vs ≥65 | 3979 | 102821 | 4838 | 131076 | 1.05 (1.01–1.09) |  |

Note: a, number of reports containing both the ICIs and hematologic and lymphatic system AEs; b, number of reports containing both the ICIs and all other adverse events (except hematologic and lymphatic system AEs); c, number of reports containing both all other drugs (except ICIs) and hematologic and lymphatic system AEs; d, number of reports containing all other drugs and all other adverse events.

CI, confidence interval; IC, information component; IC_025_, lower limit of the 95% two-sided confidence interval of the IC; IC_975_, upper limit of the 95% two-sided CI of the IC; ROR, reporting odds ratio; ROR_025_, lower limit of the 95% two-sided CI of the ROR; ROR_975_, upper limit of the 95% two-sided CI of the ROR.
